# Disordered gambling: the evolving concept of behavioral addiction

**DOI:** 10.1111/nyas.12558

**Published:** 2014-10-21

**Authors:** Luke Clark

**Affiliations:** Department of Psychology, Centre for Gambling Research, University of British ColumbiaVancouver, Canada

**Keywords:** pathological gambling, dopamine, internet gambling disorder

## Abstract

The reclassification of gambling disorder within the *Diagnostic and Statistical Manual of Mental Disorders*, Fifth Edition (DSM-5) addictions category marks an important step for addiction science. The similarities between gambling disorder and the substance use disorders have been well documented. As gambling is unlikely to exert actively damaging effects on the brain, the cognitive sequelae of gambling disorder may provide insights into addictive vulnerabilities; this idea is critically evaluated in light of recent structural imaging data. The second part of the review analyzes a fundamental question of how a behavior can become addictive in the absence of exogenous drug stimulation. The relative potency of drug and nondrug rewards is considered, alongside evidence that cognitive distortions in the processing of chance (for example, the illusion of control and the gambler's fallacy) may constitute an important added ingredient in gambling. Further understanding of these mechanisms at neural and behavioral levels will be critical for the classification of future behavioral addictions, and I consider the current research data for obesity and binge eating, compulsive shopping, and internet gaming disorder.

## Introduction

In 2013, the release of the *Diagnostic and Statistical Manual of Mental Disorders*, Fifth Edition (DSM-5) announced a major shift in the conceptualization of addiction, with gambling disorder (previously termed pathological gambling) moved from its previous home in the Impulse Control Disorders category of the DSM-III and DSM-IV, to a new location alongside the substance use disorders in a category labeled Substance-Related and Addictive Disorders.^1^ In addition to some technical changes to the diagnosis itself (omission of the illegal acts criterion, and moving the threshold to four of nine symptoms), the important step here was in the formal ratification of *behavioral addiction* as a clinical and neurobiological entity. The scientific evidence that precipitated this reorganization was diverse, including similarities between gambling disorder and substance use disorders in symptom profile (e.g., tolerance, craving, and withdrawal), comorbidities, heritability, and brain changes (including both neurocognitive testing and neuroimaging). This evidence has been reviewed extensively in previous articles^2^,^3^ and will not be revisited here.

The decision opens up several new questions, which feed back to address the nature of addiction itself. This article is structured around three questions. First, it is well recognized that chronic exposure to many drugs of abuse can exert harmful effects on the brain, and that these effects create a “chicken and egg” problem in identifying neural and psychological markers associated with addiction vulnerability.^4^,^5^ Assuming that these damaging effects are negligible in the case of gambling disorder, what insights can be gleaned from the neural or cognitive sequelae seen in a behavioral addiction? Second, various neuroscience models of addiction rely on the powerful ability of drugs of abuse to exogenously stimulate brain neurotransmitter systems, with a particular emphasis on the mesolimbic dopamine pathway.^6^ If natural rewards target the same systems—but endogenously and in a less potent manner—then how do behaviors like gambling actually become addictive? One possibility is that cognitive distortions in the processing of chance constitute a necessary added ingredient in gambling disorder. The third question notes that, at present, gambling disorder is the only behavioral addiction in the new DSM-5 category, but that other conditions have been earmarked for further research; notably internet gaming disorder, which has been listed in the DSM-5 appendices. How do data on the neurobiological foundations of gambling disorder help to inform decisions for future inclusion, and what are the main candidates?

## Neurotoxicity

The detrimental effects of various addictive drugs have been extensively documented, for example (1) longitudinal neuroimaging studies have shown that alcohol dependence is associated with progressive tissue shrinkage across multiple brain regions, concentrated on frontal and cerebellar networks,^7^ (2) postmortem studies in methamphetamine users identify histological markers of cell death in the orbitofrontal cortex,^8^ and (3) in experimental animals, short-term (2-week) cocaine regimens induce persistent changes in multiple facets of inhibitory control compared to animals treated with saline injections.^9^,^10^ Such effects are often labeled *neurotoxicity*, although given that actual cell atrophy cannot be inferred from structural neuroimaging data or cognitive sequelae, it is more conservative to regard them as neuroadaptive changes.

Certainly, it is naive to think that a behavioral addiction, such as gambling disorder should be immune to such neuroplasticity, as this is thought to be the physiological hallmark of all learning. Episodes of gambling are linked to activation of the sympathetic nervous system and cortisol release, with associated (nongenomic) changes,^11^,^12^ and gambling disorder is also reasonably comorbid with substance use disorders.^13^ Nevertheless, it seems reasonable to suppose that the actively damaging effects of addictive drugs should be absent or at least minimal in individuals with gambling disorder without such comorbidities. In the most extreme version of this argument, gambling disorder may constitute a prototypical addiction^4^ and offer a means of studying the addictive process in brains that are not disrupted by exogenous drug effects.

With this rationale in mind, neuropsychological studies have compared groups with gambling disorder and substance use disorders against nonaddicted controls, identifying impulsivity as a key shared marker. Impulsivity is a multifactorial trait characterized by unplanned responding and hasty decision making that may be unduly risky or neglect negative consequences.^14^ A nonclinical group with mild gambling disorder showed an elevation in risky decision making (impulsive choice) on the Cambridge Gamble Task, which was also seen in a second group of patients with alcohol dependence.^15^ The alcohol-dependent group showed additional deficits in response inhibition^16^ (impulsive action) and spatial working memory, which were unaffected in the gamblers, and may reflect progressive alcohol-induced changes in the lateral prefrontal cortex (PFC). It is perhaps unfortunate that the other work in this area compares gambling disorder against other substance use disorders. For example, the comparison with stimulant users may be pertinent given the physiological “high” that is also seen during gambling; in recent work, patients with gambling disorder showed greater deficits in delay discounting (another form of impulsive choice) than a cocaine-dependent group.^17^ Differences in Stroop inhibition and trait mood-related impulsivity (urgency) were present in both groups, and the cocaine users showed a selective impairment in working memory that correlated with measures of cocaine exposure.

Using functional neuroimaging, other work has examined the comparison with nicotine dependence, a drug addiction where overt neurotoxicity might be expected to be negligible. Individuals with gambling disorder and nicotine dependence showed reduced recruitment of the ventrolateral PFC by choice feedback (both gains and losses) on a reversal learning task^18^ and reduced activation of the dorsomedial PFC during a response inhibition task.^19^ Executive planning tasks dependent on the dorsolateral PFC were spared in both groups.

The pattern that emerges from these studies is one of impulsivity as a shared marker that is therefore proposed to reflect the predisposition to develop a range of addictive disorders, including gambling disorder but also conceivably other non-drug-related risky behaviors, such as risky sexual behaviors.^20^ This conclusion is substantiated by several other lines of evidence. Taking an endophenotype approach, Ersche *et al*. have compared cognitive and imaging markers in stimulant users and their unaffected first-degree relatives, showing a shared elevation of impulsivity^21^ underpinned by white matter integrity of the lateral PFC;^22^ notably, the related trait of sensation seeking was only present in the affected probands and may thus be a determinant of initial recreational engagement with drugs. Analogous studies in family members of individuals with gambling disorder are currently in progress. The children of parents with substance use disorders constitute an alternative high-risk group, and also display markers of both impulsive action^23^ and impulsive choice.^24^ Prospective designs arguably provide the most compelling evidence for premorbid vulnerability, and the small number of existing studies also point to trait impulsivity and poor self-control from as early as 3 years of age, predicting later development of gambling problems, as well as alcohol, nicotine, and marijuana misuse.^25^–^27^

But to what extent can markers seen in gambling disorder really be directly extrapolated to addiction vulnerability? Recent work has begun to examine brain structure in this condition. Two initial studies using whole-brain voxel-based morphometry in patients with gambling disorder failed to identify any regions showing significant gray matter change.^28^,^29^ By contrast, widespread reductions were observed in a matched group of heavy drinkers.^29^ However, with an *a priori* focus on the striatum and the PFC, a later study in gambling disorder detected increased gray matter volumes in both regions,^30^ and similar hypertrophy has been described as a marker of regular video game play in adolescents.^31^ Changes in white matter tracts and resting-state connectivity have also been reported in gambling disorder.^28^,^32^ A reasonable conclusion at the current time is that signs of structural brain changes can be detected in gambling disorder, but these changes appear minor in comparison to most substance addictions. What is needed next are longitudinal studies that chart the changes within a group of gamblers over time, particularly around the time of their transition into disordered and compulsive use.

## Dopamine and the brain reward system

The common action of drugs of abuse to stimulate mesolimbic dopamine transmission is a cornerstone of the modern concept of the brain reward system.^6^,^33^ Pathophysiology within this system has also emerged as central to gambling disorder, although the nuances of this disruption remain unclear. Functional magnetic resonance imaging (fMRI) studies using reward processing and decision-making tasks describe consistent abnormalities across the key nodes in this circuit in gambling disorder—the striatum, medial PFC, amygdala, and insula. However, some experiments point to hypoactivity within this system,^34^–^36^ while others indicate hyperactivity of the same regions.^37^,^38^ These two sets of data support contrasting theoretical positions (reward deficiency versus sensitization/incentive salience accounts, respectively^39^,^40^).

Although some of these discrepancies may be due to technical issues in resolving certain brain regions and the design challenges in temporally segregating selection, anticipation, and outcome periods within a trial,^40^ two exemplary recent studies provide a different perspective. Sescousse *et al*.^41^ modified the classic monetary incentive delay task^42^ to compare brain activity during both the anticipation and receipt of monetary rewards and sexual rewards (i.e., erotic images). Behavioral data (response latencies) indicated an interaction effect such that males with gambling disorder (*n* = 18) were more motivated by financial reward but less motivated by the erotic rewards. The same interaction was also expressed in the ventral striatum, which showed hypoactivity to the erotic cues. The orbitofrontal cortex in the patients with gambling disorder showed a stronger response to the monetary outcomes, and also appeared to process these financial rewards as if they were primary rewards (money is a highly ingrained conditioned reinforcer).^43^ The important message from this paper is that addictions may be associated with an imbalance between different reward types,^41^ and that the compatibility of the task reward with the abused commodity will critically determine changes in the brain reward system.^35^

The second study investigated the impact of gambling-related cues (conceptualized as Pavlovian conditioned stimuli) on decision making in gambling disorder.^44^ Gambling images (e.g., slot machines, casino photos) were presented in the background during delay-discounting choices, and the decisions were subsequently segregated for analysis on the basis of subjective craving ratings. The gamblers made more impulsive choices (of smaller-sooner rewards) in the presence of the high-craving cues, and these cues also reversed the usual pattern of subjective value coding in the midbrain and ventral striatum. It is important to point out that many fMRI studies of gambling have used abstract tasks that do not involve naturalistic cues or even real monetary rewards, which are critical influences on a gambler's decision making. It has long been noted that the irrational thoughts about chance and skill displayed by individuals with gambling disorder are greatly exacerbated by (and perhaps even restricted to) actual gambling play,^45^ and a great deal of further work is expected using the design by Miedl *et al*.

**Figure 1:**
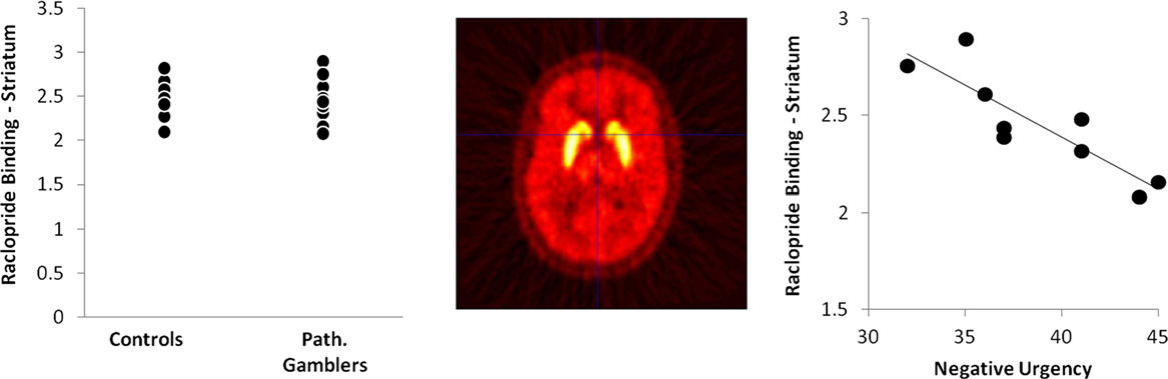
Using PET with the [^11^C]raclopride ligand, we compared baseline binding as a marker of dopamine D_2_/D_3_ receptor availability in nine males with gambling disorder against nine male healthy participants. We detected no group difference in binding potential in regions of interest encompassing the overall striatum or the limbic subdivision that includes the nucleus accumbens. A representative image from one participant (a healthy control) is shown centrally, illustrating the striatal localization of raclopride binding. Despite the absence of a group difference, raclopride binding within the gamblers was negatively related with trait-impulsivity levels, on a measure of mood-related impulsivity (urgency) that shows robust group differences between gambling disorder and controls.^85^ Data redrawn from Ref. 51.

Of course, fMRI provides only an indirect measure of dopamine transmission, and other work has used positron emission tomography (PET) with dopamine radioligands like [^11^C]raclopride. Reduced striatal dopamine D_2_ receptor binding is a robust effect in individuals with drug addictions; this has been observed across stimulant,^46^ heroin,^47^ alcohol,^48^ and nicotine dependence,^49^ as well as in preclinical models of high impulsivity.^50^ Thus, there was a strong prediction that individuals with gambling disorder should display the same effect. Surprisingly, four independent PET studies to date have failed to detect any group differences in baseline (i.e., resting) dopamine D_2_ receptor availability in gambling disorder,^51^–^54^ although individual differences were seen as functions of impulsivity markers^51^ and symptom severity^54^ (Fig.1). Returning to the first question of neurotoxicity, a plausible inference is that the effects described in drug addiction are more reflective of drug-induced changes as opposed to preexisting vulnerability. Of greatest interest, a recent study has quantified dopamine *release* in gambling disorder, using a different ligand, [^11^C] (+)-4-propyl-9-hydroxynaphthoxazine (PHNO), in combination with amphetamine challenge.^55^ Previous studies in stimulant addicts consistently reported blunted dopamine release,^56^,^57^ but in a group of 12 males with gambling disorder, dopamine release in the dorsal striatum was increased relative to healthy males. The dopamine response to amphetamine was positively predicted by the baseline PHNO binding in the substantia nigra, which is thought to specifically reflect dopamine D_3_ receptor levels, and it was also correlated with symptom severity.^55^ In summary, the PET work in gambling disorder points to clear perturbations in dopamine transmission, but this is one area where the emerging profile in gambling disorder increasingly diverges from the established picture in drug addiction.

## The relative potency of drugs of abuse

In animal models of addiction, it is often asserted that drugs of abuse target the same brain systems that mediate ordinary pleasures, but that they are considerably more potent than natural rewards at doing so. This idea is implicit in the dogmatic view that drugs of abuse hijack the ascending dopamine projection. If true, this presents a problem for the construct of behavioral addictions, which must rely on either natural or conditioned reinforcement mechanisms. If these rewards are only capable of eliciting a weak response (or at least inside the normal physiological range) within the critical neural circuitry, then behaviors like gambling may require additional ingredients in order for them to transition into a full-blown addicted state.

To what extent is this potency argument empirically supported? The key data here date back to the advent of *in vivo* microdialysis in the 1980s, at which point it became possible to measure extracellular dopamine levels in the nucleus accumbens during exposure to different rewards. Intravenous stimulants provoked at least a fivefold increase in accumbens dopamine,^58^,^59^ with intravenous heroin eliciting increases of 150–300%.^60^ In a notable study that compared this change following amphetamine and cocaine with food reward, food was associated with a 37% increase in dopamine^58^ (this was nonetheless highly significant and viewed as impressive within the natural physiological range). Similarly, copulatory behavior in sexually experienced male rats was associated with roughly a doubling of accumbens dopamine.^61^ Thus, the microdialysis studies collectively indicate greater evoked dopamine response by drugs of abuse compared to natural rewards.

Several lines of evidence with alternative methodologies call for a toning down of any qualitative difference in potency. In giving animals a lever choice between cocaine and sucrose administration, rats reliably opt for sucrose,^62^ a finding that even occurs in animals that chronically use cocaine.^63^,^64^ Thus, exogenously driven dopamine increases may be profound, but animals may nevertheless prefer natural rewards. The fact that around 10% of rodents reliably select cocaine in this paradigm also harks back to the importance of vulnerability factors like impulsivity.^63^ Other work has recorded from neurons in the ventral striatum during tasks that are reinforced with drug or nondrug rewards. Using a go/no go procedure, Carelli *et al*.^65^ observed a substantial majority of cells that responded to cues associated with either cocaine or water, but only 8% of cells responded to both (see also Ref. 66). A later study found that the proportion of ventral striatal cells responding to both cocaine and sucrose was considerably higher (50%).^67^ In those cells with the joint-firing profile, the phasic response was greater to cocaine cues than sucrose cues, but only by around 50%, which is rather less than the ballpark difference indicated by the microdialysis studies. Other work highlights full dissociations in the processing of natural rewards and drug rewards. In particular, manipulations of the subthalamic nucleus have been seen to reduce cocaine self-administration while increasing motivation to drink sucrose;^68^,^69^ it is unclear what these data might signify for the behavioral addictions, but it is important clinically as proof-of-principle data that treatments for addiction need not necessarily induce reductions in naturally rewarded behaviors.

## Addictive ingredients

An influential computational model of addiction by Redish *et al*.^70^ also gives a critical role to the fact that drug-induced stimulation of dopamine transmission is exogenous. Redish's model is grounded in seminal monkey electrophysiology work by Schultz^71^ showing that, over the course of appetitive Pavlovian conditioning, the midbrain dopamine cells initially fire to the unconditioned stimulus (US; e.g., fruit juice delivery), but as learning occurs this response tracks back to the conditioned stimulus (CS; e.g., a visual stimulus). Following learning, there is no phasic firing to the US, as there is no prediction error. These Pavlovian processes are pervasive in drug addiction (for example, cigarette packets or injection paraphernalia), and Redish proposed that, by exogenously stimulating the dopamine system, drugs of abuse continually elicit a US response, giving rise to a kind of hyper-learning about associated cues (see also Ref. 72). Data from fast-scan voltammetry substantiate this double response to both the CS and US in midbrain dopamine cells of experimental animals self-administering cocaine,^73^ although it should be noted that tests of this hypothesis in humans are more equivocal.^74^,^75^

*Prima facie*, this model is intuitively appealing, but also implies a unique capability of addictive drugs, rather than natural behaviors, to create an addicted state. As I have already discussed,^44^ comparable Pavlovian processes seem to occur in gambling behavior. In an fMRI study by van Holst *et al*.,^37^ patients with gambling disorder and healthy controls played a card-guessing game for small (€1) or large (€5) financial gains and losses. After an anticipatory delay, the financial outcome was revealed. The gamblers showed greater gain-related activity during this anticipation interval (followed by some blunting of the response to the outcomes), consistent with a response tracking from the US (the jackpots) to the CS (the spins). These learning processes were formally examined within a slot machine game, by having one group of participants practice the game extensively before scanning, and a second group who played without practice.^76^ The practiced group showed reduced striatal and amygdala activity to the jackpot wins, in combination with heightened activity to the spinning of the reels; these transitions were further moderated by trait impulsivity.

Redish *et al*.^77^ later updated their model to explicitly consider the case of gambling addiction, with two critical added features. The first is acknowledgment of the “big win” hypothesis, that many people with gambling disorder retrospectively describe receiving major payouts in the first few times that they ever gambled. In one study,^78^ 26% of a group with gambling disorder reported such a win (averaging $620) the first time that they ever gambled. These early wins constitute profound positive prediction errors that will activate the neural machinery of reinforcement learning. The second feature is the asymmetry in the temporal-difference learning model between appetitive and aversive outcomes. Financial gains (positive prediction errors) promote straightforward learning acquisition, but financial losses (negative prediction errors) do not trigger simple unlearning, as illustrated by reinstatement phenomena following extinction. Rather, financial losses may promote specific instances of learning that Redish calls “state splitting,”^77^ and that gambling researchers recognize as “hindsight bias,” the explaining away of losses in a manner that does not erode the player's belief in his/her ability to win.^79^

In summary, it is incontrovertible that drugs of abuse effectively stimulate the mesolimbic dopamine system, and that this action is common to both natural rewards and gambling outcomes as well. The evidence for increased potency of drug rewards varies substantially across different neuroscience methods. Even if one accepts the microdialysis data for the size of the dopamine response to drugs of abuse, it is still not clear whether this potency shapes actual choice behavior. Such massive drug-induced reward may be too intense for many individuals.^80^ A conservative conclusion is that drugs of abuse are quantitatively, rather than qualitatively, more potent than natural rewards,^67^ but the basic fact that the dopamine stimulation is exogenous in drug addiction may by itself introduce some unique considerations, as in the Redish computational model. These latter alternatives raise the possibility that behavioral addictions require an added ingredient. The modification of the Redish model to accommodate gambling addiction gives two clues as to the nature of these ingredients. The first is decision uncertainty, given that learning from prediction errors only occurs in uncertain environments. The second is the potential for bivalent outcomes (i.e., gains and losses). In the next section, I will elaborate on some further properties of gambling games that may render them addictive, drawing upon the evidence for gambling-related cognitive distortions under conditions of chance.

## Gambling-related cognitive distortions

A standard definition of gambling refers to an individual risking something of value (i.e., accepting a cost) on the uncertain prospect of a larger reward.^81^ It is often noted that this definition encompasses many other aspects of human behavior,^82^ as well as the everyday decisions of virtually all nonhuman animals in foraging for food, seeking shelter, and avoiding predators.^83^ However, the element of chance in gambling games raises some unique problems for cognition. Most environments in the natural world are probabilistic, and the neural machinery of reinforcement learning has evolved with exquisite sensitivity to these contingencies. By contrast, gambling games are mostly entirely random (slot machines, lotteries, roulette) or involve a modest (and opaque) degree of skill (blackjack, sports betting).

Humans display a number of systematic errors in processing under conditions of chance, which come to the fore in gambling games and are known as gambling-related cognitive distortions.^45^,^84^ There is emerging evidence that individuals with gambling disorder may be more prone to these gambling distortions than the general population; much of this evidence comes from self-report measures like the Gambling-Related Cognitions Scale^85^ or the Gambling Beliefs Questionnaire,^86^ which ask about a variety of specific beliefs and biases, and yield elevated scores in groups with gambling disorder.^87^–^89^

Two common classes of distortion that have been widely studied in the laboratory are the *illusion of control* and the *gambler's fallacy*. The illusion of control refers to irrelevant features of a game that create a sense that one is developing some kind of skill over an outcome that is in fact determined by chance alone.^90^,^91^ Gamblers are more risky and more confident when they can make an irrelevant choice (e.g., choosing their lottery numbers) or when they can exert instrumental action (e.g., throwing dice or a roulette ball).^92^–^94^ Numerous other features such as the presence of a competitor or the availability of background information (like recent form guides in sports betting) may feed into the same belief. In a task where participants estimated their degree of control over a noncontingent response–outcome association, individuals with gambling disorder showed higher perceived control than healthy participants.^95^

The gambler's fallacy occurs when observing sequences of random outcomes, like coin tosses or red/black outcomes in roulette.^96^ Following a run of the same outcome (e.g., four successive roulette spins landing on red), players typically predict that the other outcome (i.e., black) will occur next. Most psychological accounts of the gambler's fallacy refer to the *law of small numbers*, that people expect small fragments from a distribution to be representative of the distribution itself, whereas in reality, runs and streaks are fairly common in random sequences. The gambler's fallacy is described in choosing lottery numbers^97^ and in casino data for roulette.^98^ As preliminary evidence that this effect is linked to gambling disorder, a higher level of gambling distortions in students predicted gambler's fallacy decisions,^99^ and at-risk gamblers preferred a slot machine simulation that delivered more “clumpy” outcomes.^100^

Other specific phenomena within gambling games can be considered under the rubric of these two effects. Most forms of gambling deliver *near misses*, outcomes that are perceived as having been close to a win, but that are in fact objective losses. Games that deliver a moderate rate of near misses are played for longer than machines that deliver none (or a low rate),^101^,^102^ and on subjective ratings, near misses are perceived as more aversive than complete misses, but increase the desire to continue the game.^93^ Given that near misses in skill-based games (e.g., soccer, archery) convey useful signals of skill acquisition, near misses may fuel the illusion of control.^93^,^103^ They may also feed into the processing of outcome sequences, by breaking up a perceived streak of losses.^104^,^105^

**Figure 2:**
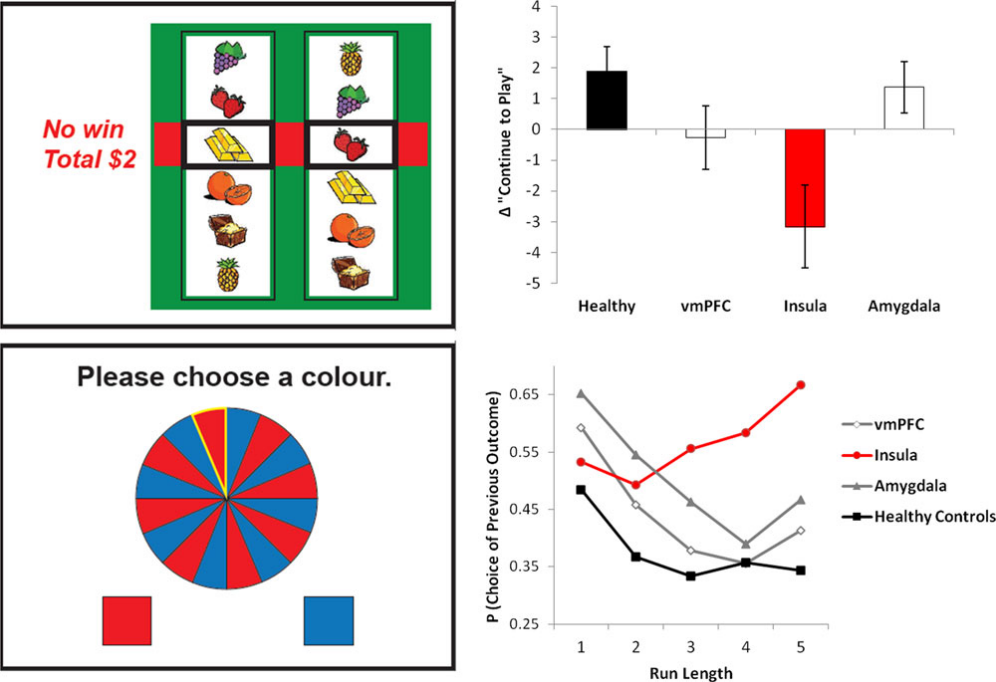
Effects of insula damage on gambling-related cognitive distortions. The slot machine task (top left) displayed two reels; one icon was selected on the left reel, and the right reel then spun and stopped to a standstill. Matching icons delivered wins; if the right reel landed one position from a match (as shown), this was considered a near miss. After each outcome, participants rated their motivation to continue to play the game. The bar chart (top right) displays this motivational ratings following near misses, compared to full misses (which do not land adjacent to the pay line). The typical motivational response to near misses (see also Refs. 93, 104, and 111) was inverted in the group with insula damage. In the roulette task (bottom left), participants made consecutive red/blue predictions on a random wheel. In healthy participants, the likelihood of choosing either color decreased linearly as a function of the preceding run length of that color; this negative recency is the gambler's fallacy effect. As for the slot machine task, the gambler's fallacy was abolished in the group with damage to the insula region. Data redrawn from Ref. 107.

Despite their objective status as losses, near misses have been reliably shown to increase the neural signal in brain reward circuitry, compared to full misses.^76^,^93^,^106^ One region that is particularly sensitive to this effect is the anterior insula. This region showed overlapping activity to wins and near misses:^93^ the insula response to the near misses correlated with trait susceptibility to gambling distortions,^93^ and in a magnetoencephalography study, the signal extending from the right orbitofrontal cortex to the insula was associated with severity of gambling disorder.^106^ Nonetheless, neuroimaging is fundamentally a correlational technique, and to convincingly demonstrate the critical role for the insula region, my colleagues and I examined neurological cases with selective damage to the insula, as well as groups with lesions to the ventromedial PFC and the amygdala and healthy participants.^107^ The participants performed two gambling tasks (Fig.2): a simplified slot machine game that delivered wins, near misses, and full misses; and a roulette game to look at choice behavior following red/black runs of varying length. Most groups showed enhanced motivation to play following near misses (on a subjective rating) and displayed the gambler's fallacy in their choice on the roulette task; both effects were selectively abolished in the group with insula pathology. These data provide evidence for the causal involvement of the insula in two classic cognitive effects in gambling, and generate a prediction that patients with gambling disorder will show overrecruitment of the insula region, rendering them more vulnerable to these cognitions.

The precise role that the insula plays during gambling remains unclear. One dominant account of insula function describes its role in interoception:^108^ the detection and awareness of bodily, visceral states. It is pertinent that gambling is intensely physiologically arousing.^109^,^110^ Indeed, near misses were previously seen to drive a phasic arousal signature (skin conductance, heart rate change) in the slot machine task.^111^ Within the broader context of the addictions, the insula is activated by multisensory drug cues and craving states in cue–reactivity designs.^112^ A previous neuropsychological study of smokers who experienced neurological damage described a spontaneous cessation of smoking following insula injury, seemingly linked to an abolition of their urges to smoke.^113^

The classic neuropsychological model of addictions by Jentsch and Taylor^114^ highlights the balance between a subcortical *accelerator* system (comprising the nucleus accumbens and amygdala) and a prefrontal cortical *braking* system. This has much in common with classic dual-process accounts of decision making.^115^ In the transition into drug addiction, the reflexive system becomes sensitized through learning processes, while the prefrontal brake is concurrently eroded by the damaging effects of the drugs. Later extensions of this model usefully separate the substrates for initial intoxication and subsequent craving, and point out the involvement of the hippocampus in holding drug-related memories.^116^,^117^ However, these models have not generally recognized the insula as an important node. While the insula may be easily grafted onto a neural diagram for the slightly nebulous concept of craving, a recent model^118^ places the interoceptive functions of the insula as a thermostat, in a three-system model where the insula represents a gateway between the subcortical reward system and the prefrontal system responsible for decision making and inhibitory control. By emphasizing the interoceptive aspects of addictions,^119^,^120^ some new avenues for treatment may also be indicated, such as physiological regulation on the basis of biofeedback or meditative or mindfulness-based techniques.

## Future behavioral addictions

A recurrent concern with the concept of behavioral addiction is the slippery slope: how do we avoid everyday passions being gradually pathologized as addictions? Golfers, for example, are a group who spend a great deal of time engaged in their hobby, invest heavily in new equipment and course fees, and probably experience some preoccupation and discomfort when forced to abstain from golf for a week or two.^121^ As golfers rarely seek treatment for their (golfing) problems, these concerns can be disregarded as clinically irrelevant, but if such behaviors are judged to cause a clinically significant impact on daily functioning (e.g., fulfilling occupational or family commitments), then some argue that it is reasonable and appropriate to label them as addictions (an alternative term such as *excessive appetites* may be preferred within this framework^122^). In the case of gambling disorder, the negative consequences are overt and manifold. Debt is a pervasive theme with undeniable consequences for financial stability and family integrity. The previous sections provide an alternative way to consider this, in terms of underlying psychological and neural mechanisms. Certainly, decisions regarding the classification of future behavioral addictions will require detailed neural and behavioral analyses of candidate disorders. Moreover, if choice uncertainty or cognitive distortions play a key role in driving the neural circuitry that ordinarily underpins reinforcement learning and compensating for the lack of exogenous drug stimulation, then it is possible that only a finite number of behaviors will have the capability to be addictive.

### Obesity and binge eating

One of the more controversial arguments has been in considering obesity from the perspective of food addiction. As support for this model, drug self-administration protocols in experimental animals show comparable phenomena (e.g., escalation, cross-sensitization) for food rewards (either sucrose or high-fat foods).^64^ Using PET imaging, reduced striatal dopamine D_2_ receptor binding was also described in nondrug users with high body mass index.^123^ Functional MRI investigations in obese individuals show a similar profile to the studies in drug addiction and gambling disorder. For example, Stice *et al*.^124^ looked at brain responses to the anticipation and receipt of milkshake rewards in adolescent girls with and without obesity: the obese group showed a heightened somatosensory response to the anticipation of food intake, coupled with a blunting of the caudate response to the actual consumption. This effect is complementary to the Pavlovian mechanisms described in gambling,^37^ and these results in obesity were further moderated by dopaminergic gene polymorphisms also linked to addiction vulnerability.^125^ There is increasing recognition that obesity is multiply determined, and that an addictions lens may be best suited to a subgroup with binge eating behaviors.^126^ In a genotyping study, the frequencies of both opioid and dopamine polymorphisms differed significantly between obese participants with and without binge eating behavior.^127^ From a decision-making perspective, obesity and binge eating can clearly be conceptualized as a persistent bias toward an option that offers immediate gratification with long-term negative consequences (for body shape and physical health). At the same time, these overeating phenotypes do not evidently involve the distortions in prediction-error signaling or deficits in the processing of chance that are described for gambling disorder.

### Compulsive shopping

For compulsive shopping, the major link to the neurobiology of drug addiction comes from Parkinson's disease, where this syndrome can appear alongside gambling disorder and hypersexuality in a constellation of reward-driven, impulsive behaviors that are seen as occasional side effects of dopamine-agonist medications.^128^ Voon *et al*.^129^,^130^ administered dopamine agonists to a group of Parkinson's patients with this syndrome (a mixture of gamblers and compulsive shoppers) and Parkinson's controls. Dopamine agonists increased gain-related prediction-error signals in the ventral striatum in the group with impulse-control disorders, in the opposite direction to the effect observed in Parkinson's controls.^129^ A similar opposing effect was observed on a gambling task where loss aversion was insensitive to dopaminergic medication, but risk taking to gain prospects was selectively heightened in the group with impulsive disorders.^130^ At the present time, very little neuroscientific research is available on this condition outside of the context of Parkinson's disease. Recent work has found that trait-reward sensitivity predicted compulsive-buying tendencies differentially from either depression or obsessive–compulsive disorder.^131^ In a cognitive study of a group who were not treatment seeking but met clinical criteria, the compulsive shoppers reported a reduced sensitivity to winning probabilities on the Cambridge Gamble Task, as well as working-memory and response-inhibition deficits.^132^ Perhaps surprisingly, this is more comparable to the profile seen in severe alcohol dependence than gambling disorder.^15^ Much as for obesity, it is arguably facile to consider compulsive shopping as a persistent choice of an immediate reward (i.e., buying an attractive item) with long-term negative consequences (i.e., for one's bank balance and self-esteem), but it is unclear whether further parallels exist in the processing of choice uncertainty and psychological distortions, and further work is needed to evaluate this claim.

### Internet gaming disorder

The most likely candidate to join gambling disorder as a behavioral addiction at the current time is *internet gaming disorder*, which has emerged recently from the umbrella term i*nternet addiction*. Affected individuals spend several hours each day playing video games—typically massively multiplayer online role-playing games (MMORPGs) like World of Warcraft—with established negative consequences in terms of academic performance and social functioning.^133^ Research into the syndrome has been somewhat hampered by a lack of consensus on its assessment, with different groups modifying drug-use or problem-gambling screens, focusing on general internet use or video game play specifically, and employing different thresholds for a diagnosis. As a result, prevalence estimates vary widely, but a recent consensus report^134^ has established a nine-item international instrument that will hopefully rectify this concern.

The cognitive and neurobiological data on internet gaming is growing at a rapid rate. A classic raclopride–PET study detected striatal dopamine release as healthy participants played a primitive video game,^135^ and reductions in striatal D_2_ binding have been described in a small study in men being treated for internet addiction.^136^ Cognitive and functional MRI studies have found evidence of cue reactivity,^137^,^138^ with game screenshots driving signal changes in the medial PFC. The syndrome is clearly associated with trait impulsivity,^139^ and cognitive impairments are described on tasks of impulsive choice and impulsive action, similar to gambling disorder.^140^,^141^ Video game play is also associated with substantial physiological arousal,^142^ similar to gambling.

The Redish *et al*. model^77^ for gambling addiction can be extended in a straightforward manner to video game play. Actions within a video game generate bivalent outcomes, and it is self-evident that humans will work to achieve symbolic gains and avoid symbolic losses, in much the same way as for monetary outcomes. The game environment is uncertain, with rewards delivered on an unpredictable variable-ratio schedule of reinforcement, which is identical to gambling. In fact, the structural characteristics of video games lend themselves to a very similar analysis to gambling games.^143^ One notable difference is the unequivocal skill dimension in video games, such that performance improves with practice. However, as games typically get progressively harder with practice, the rewards within the game remain essentially unpredictable. It is in fact typical for several reinforcement schedules to overlap within a game (e.g., killing the monster, finishing the level, and accessing special features like hidden rooms). For video gamers who also gamble, a failure to differentiate skill and chance environments may pose a particular risk for illusory control, and there is preliminary evidence that regular gamers may be more disposed to perceive skill in gambling tasks.^144^ Gambler's fallacy beliefs may be relevant to continued play following long runs of losses.^145^ Modern MMORPGs also contain some unique features that are absent from either traditional computer games or gambling. One is the avatar: players play as a consistent character who gradually develops within the game environment across many sessions. The second is the multiplayer component, which means that the game continues while any individual player is offline, which could foster checking behaviors or counterfactual thoughts. As such, internet gaming is a behavior that lends itself to evaluation in terms of discrete psychological properties and comparisons with the cognitive effects of gambling. While the field is too premature to allow any strong conclusions, this is set to be a fruitful line of inquiry.

## Conclusions

Behavioral addiction is a field that has come of age recently, both in the sense of the formal ratification of the construct as a clinical entity in the DSM-5 and scientifically in terms of a credible evidence base that has begun to elucidate the underlying psychological and neurobiological mechanisms. Much of this research has been directed at gambling disorder, which is treated here as the blueprint for the rest of the field. Despite the presumed absence of damaging (putatively neurotoxic) effects, gambling disorder displays a clear neurocognitive profile with evidence of impulsive choice (risky decision making and preference for immediate rewards) even in relatively less severe cases, coupled with wider disruptions in executive control in the most severe cases. These indications of impulsivity chime with other lines of evidence for a cognitive endophenotype across the addictive disorders.

Nevertheless, it is likely that chronic engagement in gambling will induce neuroadaptive effects,^64^ and future longitudinal work is needed to characterize the degree of structural and functional change within gamblers, particularly around the time of onset of pathological behavior. Some methodological difficulties recur across much of the cognitive and neuroscience research on gamblers’ decision making: many gambling tasks do not involve ecologically valid stimuli or genuine monetary rewards, and even when real money is available, it is questionable whether the sums used have similar subjective value to seasoned gamblers and nongamblers. It should also be recognized that gambling disorder is not a homogenous condition and is unlikely to arise from a single etiological mechanism. Within the influential pathways model,^146^ impulsivity and neurocognitive sequelae are proposed to only be present in one subtype of gambler (with a high rate of overlap with attention deficit hyperactivity disorder and antisocial personality disorder). The two alternative pathways to gambling disorder are through straightforward behavioral conditioning (e.g., a big win in initial gambling experiences) and through negative reinforcement to alleviate depression or anxiety. It is unclear whether neurobiological changes will cut across these subtypes (as a final common pathway) or be linked to one specific subtype, and few studies have adequate sample sizes to investigate these sources of heterogeneity.

Neurobiological studies also point to some emerging differences between gambling disorder and the drug addictions, which are most pronounced in the PET imaging data for dopamine disruption. Much of the current medical understanding of addiction is based on the common action of drugs of abuse to stimulate mesolimbic dopamine transmission. While dopamine is also heavily implicated in gambling disorder, the pathophysiology appears quite different. A more complete understanding of these differences will likely be as important for the conceptualization of behavioral addictions as the recognition of the similarities with drug addiction.

I have reviewed evidence for the widely held notion that drugs of abuse are qualitatively more potent at driving the dopamine system than natural rewards like food and sex. The significance of these data from experimental animals to the processing of monetary rewards in humans is not entirely clear; money is a complex conditioned reinforcer, and even animal models of gambling behavior utilize food rewards and time-out penalties.^147^ Some fMRI data suggest that patients with gambling disorder may process monetary rewards as if they were primary rewards.^41^ The empirical evidence for the potency argument is actually patchy, with some evidence that drug rewards are quantitatively more effective, and other evidence for domain independence of drug and nondrug rewards. Conceptually, the greatest challenge for the behavioral addictions may be in accommodating the exogenous effects of drugs of abuse to interfere with dopaminergic reinforcement learning.

In revisiting the Redish model,^70^ I have argued that gambling may have certain psychological properties that underlie its addictive potential. These properties may be as straightforward as bivalent outcomes occurring in an uncertain environment, or may be more aligned with the cognitive distortions that arise under conditions of chance, like the illusion of control and the gambler's fallacy. This perspective provides a framework for considering other candidate behavioral addictions like obesity/binge eating, compulsive shopping, and internet gaming disorder. It is possible to consider any excessive behavior as a deficit in decision making, based on the recurrent choice of immediate gratification despite its long-term negative consequences. However, this argument borders on the circular. Future decisions regarding the classification of new behavioral addictions will require careful analysis at the neural and behavioral level. The psychological properties that appear to underlie the addictive capacity of gambling can be extended in a straightforward manner to video game play, but less easily to overeating and compulsive shopping. From identifying the critical psychological variables, we can then understand how these behaviors become habitual and erode self-control.
